# Trajectories of chronic multimorbidity patterns in older patients: MTOP study

**DOI:** 10.1186/s12877-024-04925-2

**Published:** 2024-05-30

**Authors:** Marina Lleal, Montserrat Baré, Susana Herranz, Josefina Orús, Ricard Comet, Rosa Jordana, Marisa Baré

**Affiliations:** 1 Clinical Epidemiology and Cancer Screening Department, Parc Taulí Hospital Universitari, Institut d’Investigació i Innovació Parc Taulí (I3PT-CERCA), Universitat Autònoma de Barcelona, Sabadell, Spain; 2 Department of Paediatrics, Obstetrics and Gynaecology, Preventive Medicine and Public Health, Autonomous University of Barcelona (UAB), Bellaterra, Spain; 3 Research Network on Chronicity, Primary Care and Health Promotion (RICAPPS), Instituto de Salud Carlos III, Madrid, Spain; 4 Creu Alta Primary Care Centre, Institut Català de la Salut, Sabadell, Spain; 5 Acute Geriatric Unit, Centre Sociosanitari Albada, Parc Taulí Hospital Universitari, Institut d’Investigació i Innovació Parc Taulí (I3PT-CERCA), Universitat Autònoma de Barcelona, Sabadell, Spain; 6 Cardiology Department, Parc Taulí Hospital Universitari, Institut d’Investigació i Innovació Parc Taulí (I3PT-CERCA), Universitat Autònoma de Barcelona, Sabadell, Spain; 7 Internal Medicine Department, Parc Taulí Hospital Universitari, Institut d’Investigació i Innovació Parc Taulí (I3PT-CERCA), Universitat Autònoma de Barcelona, Sabadell, Spain; 8 Can Rull– Can Llong Primary Care Centre, Parc Taulí Hospital Universitari, Institut d’Investigació i Innovació Parc Taulí (I3PT-CERCA), Universitat Autònoma de Barcelona, Sabadell, Spain

**Keywords:** Multimorbidity, Cluster analysis, Trajectories, Longitudinal study, Older patients, Ageing, Chronic conditions

## Abstract

**Background:**

Multimorbidity is associated with negative results and poses difficulties in clinical management. New methodological approaches are emerging based on the hypothesis that chronic conditions are non-randomly associated forming multimorbidity patterns. However, there are few longitudinal studies of these patterns, which could allow for better preventive strategies and healthcare planning. The objective of the MTOP (Multimorbidity Trajectories in Older Patients) study is to identify patterns of chronic multimorbidity in a cohort of older patients and their progression and trajectories in the previous 10 years.

**Methods:**

A retrospective, observational study with a cohort of 3988 patients aged > 65 was conducted, including suspected and confirmed COVID-19 patients in the reference area of Parc Taulí University Hospital. Real-world data on socio-demographic and diagnostic variables were retrieved. Multimorbidity patterns of chronic conditions were identified with fuzzy c-means cluster analysis. Trajectories of each patient were established along three time points (baseline, 5 years before, 10 years before). Descriptive statistics were performed together with a stratification by sex and age group.

**Results:**

3988 patients aged over 65 were included (58.9% females). Patients with ≥ 2 chronic conditions changed from 73.6 to 98.3% in the 10-year range of the study. Six clusters of chronic multimorbidity were identified 10 years before baseline, whereas five clusters were identified at both 5 years before and at baseline. Three clusters were consistently identified in all time points (*Metabolic and vascular disease, Musculoskeletal and chronic pain syndrome, Unspecific*); three clusters were only present at the earliest time point (*Male-predominant diseases*, *Minor conditions and sensory impairment*, *Lipid metabolism disorders*) and two clusters emerged 5 years before baseline and remained (*Heart diseases* and *Neurocognitive*). Sex and age stratification showed different distribution in cluster prevalence and trajectories.

**Conclusions:**

In a cohort of older patients, we were able to identify multimorbidity patterns of chronic conditions and describe their individual trajectories in the previous 10 years. Our results suggest that taking these trajectories into consideration might improve decisions in clinical management and healthcare planning.

**Trial registration number:**

NCT05717309.

**Supplementary Information:**

The online version contains supplementary material available at 10.1186/s12877-024-04925-2.

## Background

Population ageing is accelerating in Europe, both in number and proportion of older individuals, as the old-age dependency ratio — i.e. population over 65 relative to population aged 15–64— is expected to continue rising. Therefore, European countries face major challenges to guarantee that their healthcare systems are prepared to tackle this demographic shift [1, 2]. 

One of these growing challenges is the clinical management of patients with multimorbidity. Multimorbidity, usually defined as the presence of two or more chronic conditions, increases with age as chronic conditions accumulate and is associated with complex treatments lacking evidence, a greater use of health resources and a lower quality of life [3–5]. Older patients with multimorbidity are excluded from clinical trials and there are few guidelines with specific recommendations for these patients [6–8]. All in all, multimorbidity poses a challenge for healthcare professionals and systems. Therefore, conducting research on how to improve multimorbid patient care in settings that have traditionally focused on single diseases should be considered a priority [9, 10].

Along these lines, recommendations have been issued to consider all chronic conditions at the same time in order to provide better patient-centred care [10, 11]. Therefore, new alternative, more comprehensive definitions of multimorbidity are being proposed, based on the hypothesis that some chronic conditions non-randomly co-occur giving rise to multimorbidity patterns. In order to obtain a thorough definition of multimorbidity patterns, detailed medical information is required. In this sense, the use of real-world data (RWD) provides valuable, readily available, large datasets [12]. Thus, in recent years, evidence has been accumulating in regards to the existence of such comprehensive multimorbidity patterns [13–18]. In fact, several patterns have already been associated with outcomes such as lower function, higher presence of adverse drug reactions, higher healthcare utilisation, poor prognosis or higher mortality [19–24]. Consequently, identifying multimorbidity patterns might aid in the development of new strategies and guidelines focusing on the most appropriate actions according to each patient profile.

Furthermore, it might be important to consider that multimorbidity profiles of each patient may progress or change over time, forming different multimorbidity trajectories. However, there are few works that have described the progression of these patterns over time [25]. Thus, developing a better knowledge of the transition pathways between multimorbidity patterns could detect profiles of patients with similar characteristics and risks who could benefit from improved, more personalized health care. Likewise, certain trajectories or belonging to certain patterns of multimorbidity over time could be associated with different prognoses or outcomes (quality of life, severity, mortality). This could allow to predict and plan for future actions, and to identify future relevant results or prognoses more accurately.

In addition, a deeper understanding of how multimorbidity develops in older adults would be desirable to plan for and deliver more appropriate care. It may also allow for the identification of targets as well as the development of programs and interventions aimed at minimising the progression and impact of multimorbidity on more distal outcomes. Therefore, these knowledge gains may guide administrators and policy makers in resource allocation. All in all, identifying multimorbidity patterns of chronic conditions and their trajectories over time might help individual patients as well as entire healthcare systems.

In this context, we developed the MTOP (Multimorbidity Trajectories in Older Patients) study, which aims to identify multimorbidity patterns of chronic conditions in a cohort of older patients, as well as their progression and trajectories in the previous 10 years.

## Methods

### Study design and cohort

The MTOP study is a retrospective observational study using RWD provided by the Agency for Health Quality and Assessment of Catalonia (AQuAS) in the framework of the Public Data Analysis for Health Research and Innovation Program (PADRIS). The study cohort is based on a cohort from a previous study on multimorbidity clusters, called MRisk-COVID study [19]. The MRisk-COVID study included 14,286 patients of a region in the Northeast of Spain (Vallès Occidental est, Catalonia), which were either confirmed or suspected COVID-19 cases from 27th February to 15th June 2020. The MRisk-COVID data was deemed suitable to address the aim of the MTOP study, as it provided readily available data on longitudinal chronic morbidity in a cohort of older patients. Patients aged > 65 years were selected, resulting in a cohort of 3988 individuals.

The study was approved by the Ethics Committee (CEIm) of the Parc Taulí University Hospital (reference 2022/5051), which waived the requirement of informed consents due to the epidemiological nature of the study and the use of anonymized data.

### Data processing and linkage

Demographic data (sex and age) and clinical data were obtained from the Shared Clinical History of Catalonia (HC3). In order to reduce patient re-identification risk, as part of the privacy policy of PADRIS, age was provided in categorized quinquennial groups, and age groups with high re-identification risk were excluded (women aged > 95 and men aged > 90).

Clinical data comprised clinical records of all primary healthcare centres in Catalonia. The provided records covered from 1930 to 2020 and encompassed all diagnoses, coded using the ICD-10-CM diagnostic system [26]. Three time points were established: 2020 (baseline, representing the time of data extraction), 2015 (5 years before) and 2010 (10 years before), and active diagnoses at each time were collected.

Data compilation, processing and statistical analysis were performed using R v3.6.0 [27].

### Multimorbidity cluster analysis

To identify chronic multimorbidity patterns, three steps were performed: identification of chronic conditions, complexity reduction of chronic conditions, and cluster analysis of patients based on these selected features. The following analyses were performed independently for each of the three selected time points.

Firstly, the identification of chronic conditions for inclusion in the analyses was carried out using the Chronic Condition Indicator software v.2021.1 [28]. This tool allows for the classification of all ICD-10-CM diagnosis codes into four categories: “Chronic condition” (value C), “Acute condition” (value A), “Both a chronic and acute condition” (value B), and “Not applicable, code cannot be used to identify a chronic or acute condition” (value N). All diagnoses with values C or B were selected. Then, these selected diagnoses were classified and grouped using the Clinical Classification Software Refined v.2021.1 [28], which allocates specific diagnoses into general chronic condition categories. This step was performed in order to reduce the number of variables for the cluster analysis and increase statistical power, while at the same time collapsing highly similar diagnoses to avoid unnecessary fragmentation. After that, chronic conditions were filtered by > 2% prevalence in order to reduce statistical noise. Only patients with two or more chronic conditions were included, regardless of the presence of acute conditions.

Due to the large number of chronic conditions, a dimension reduction was performed by Multiple Correspondence Analysis. Optimal number of dimensions was determined by the elbow criteria in the scree plot.

Soft clustering analysis was performed using the fuzzy c-means algorithm [29]. Given that it is a stochastic method; a hundred iterations were performed in order to obtain reproducible results. Several values of fuzziness (m) were tested (m = 1.1, 1.2, 1.4, 1.5, 2, 4) and the optimal m = 1.1 was estimated by the mean calculation of five indexes: Calinski–Harabasz, Partition coefficient, Partition entropy, Fukuyama, and Xie-Beni. Also, several values of the number of clusters (C = 4, 5, 6 and 7) were tested. The final C for each time point was reached through statistical criteria and consensus among 6 medical doctors from different specialties. This agreement was achieved following an independent voting process. Consensus was defined when one of the options reached a majority of votes. Cases with lack of consensus or similar voting count were meant to be decided through a clinical debate session, however this turned out not to be necessary.

After establishing multimorbidity clusters, three indicators were calculated for each chronic condition: prevalence within the cluster (%), observed/expected (O/E) ratio (prevalence in the cluster / total prevalence) and exclusivity (patients with the condition in the cluster / total of patients with the condition). Finally, a descriptive label was agreed upon and assigned to each cluster to summarize their over-represented chronic conditions and facilitate clinical interpretation.

### Statistical analysis

Each patient was allocated to the most probable cluster at each of the time points. Percentages of patients in each cluster were calculated, including patients with zero or one chronic conditions, which were allocated to a “No multimorbidity” group. Then, trajectories of each patient through the three time points were established. Percentages of patients for all possible trajectories were calculated for the three time points or pairwise (10 years before vs. 5 years before, 5 years before vs. baseline).

A stratified descriptive analysis was conducted by sex and two age groups, thereby obtaining four different patient datasets (groups of 66–80 and > 80 years, separated into male and female individuals) and plotting their trajectories separately. Age cut-off was set at 80 years to define very old individuals [30].

## Results

A total of 3988 patients aged 65 or older were included in the study, with 58.9% females and 42.4% very old individuals (aged > 80). At baseline, 98.3% of patients had multimorbidity as defined by the presence of two or more chronic conditions, whereas 5 years before it was 92.6% and 10 years before, 73.6%. The median number of chronic conditions per patient was 9 at baseline, 6 five years before and 3 ten years before baseline. Cohort characteristics are described in the first column of Table 1.

**Table 1:** Descriptive statistics of the cohort. First column contains the figures for the whole cohort, the rest of columns describe patients’ characteristics according to their assigned chronic multimorbidity clusters at the three defined time points (10 years before, 5 years before and baseline).

		Total	10 years before							5 years before						Baseline					
			Metabolic and vascular diseases	Male-predominant diseases	Minor conditions and sensory impairment	Musculoskeletal and chronic pain syndrome	Lipid metabolism disorders	Unspecific	No multimorbidity	Heart diseases	Metabolic and vascular diseases	Neurocognitive	Musculoskeletal and chronic pain syndrome	Unspecific	No multimorbidity	Heart diseases	Metabolic and vascular diseases	Musculoskeletal and chronic pain syndrome	Neurocognitive	Unspecific	No multimorbidity
n (%)		3988	168 (4.2)	364 (9.1)	370 (9.3)	426 (10.7)	472 (11.8)	854 (21.4)	1334 (33.5)	382 (9.6)	458 (11.5)	559 (14.0)	748 (18.8)	1479 (37.1)	362 (9.1)	447 (11.2)	534 (13.4)	715 (17.9)	903 (22.6)	1306 (32.7)	83 (2.1)
Nº chronic conditions, median (IQR)		9 (6–12)* 6 (4–8)** 3 (1–5)***	5 (4–7)	4 (3–5)	5 (4–7)	4 (3–6)	4 (3–5)	3 (2–5)	1 (0–1)	9 (7–11)	8 (6–10)	6 (5–8)	7 (6–9)	4 (3–6)	1 (0–1)	13 (11–16)	11 (9-13.75)	11 (9–13)	9 (7–12)	6 (4–8)	1 (0–1)
Sex	Female	2348 (58.9)	96 (57.1)	44 (12.1)	291 (78.6)	355 (83.3)	298 (63.1)	528 (61.8)	736 (55.2)	252 (66.0)	197 (43.0)	427 (76.4)	668 (89.3)	611 (41.3)	193 (53.3)	318 (71.1)	137 (25.7)	646 (90.3)	683 (75.6)	522 (40.0)	42 (50.6)
	Male	1640 (41.1)	72 (42.9)	320 (87.9)	79 (21.4)	71 (16.7)	174 (36.9)	326 (38.2)	598 (44.8)	130 (34.0)	261 (57.0)	132 (23.6)	80 (10.7)	868 (58.7)	169 (46.7)	129 (28.9)	397 (74.3)	69 (9.7)	220 (24.4)	784 (60.0)	41 (49.4)
Age group at baseline	66–70	781 (19.6)	32 (19.0)	45 (12.4)	33 (8.9)	83 (19.5)	86 (18.2)	135 (15.8)	367 (27.5)	36 (9.4)	86 (18.8)	65 (11.6)	142 (19.0)	327 (22.1)	125 (34.5)	33 (7.4)	105 (19.7)	145 (20.3)	69 (7.6)	388 (29.7)	41 (49.4)
	71–75	798 (20.0)	32 (19.0)	74 (20.3)	64 (17.3)	89 (20.9)	102 (21.6)	144 (16.9)	293 (22.0)	50 (13.1)	89 (19.4)	62 (11.1)	175 (23.4)	346 (23.4)	76 (21.0)	56 (12.5)	108 (20.2)	188 (26.3)	94 (10.4)	336 (25.7)	16 (19.3)
	76–80	720 (18.1)	36 (21.4)	89 (24.5)	54 (14.6)	86 (20.2)	77 (16.3)	164 (19.2)	214 (16.0)	61 (16.0)	92 (20.1)	88 (15.7)	144 (19.3)	277 (18.7)	58 (16.0)	79 (17.7)	110 (20.6)	151 (21.1)	132 (14.6)	235 (18.0)	13 (15.7)
	81–85	909 (22.8)	39 (23.2)	93 (25.5)	112 (30.3)	93 (21.8)	112 (23.7)	232 (27.2)	228 (17.1)	114 (29.8)	126 (27.5)	144 (25.8)	171 (22.9)	300 (20.3)	54 (14.9)	153 (34.2)	150 (28.1)	128 (17.9)	253 (28.0)	215 (16.5)	10 (12.0)
	86–90	605 (15.2)	24 (14.3)	59 (16.2)	84 (22.7)	57 (13.4)	68 (14.4)	133 (15.6)	180 (13.5)	102 (26.7)	51 (11.1)	134 (24.0)	92 (12.3)	186 (12.6)	40 (11.0)	106 (23.7)	56 (10.5)	81 (11.3)	250 (27.7)	109 (8.3)	3 (3.6)
	91–95	175 (4.4)	5 (3.0)	4 (1.1)	23 (6.2)	18 (4.2)	27 (5.7)	46 (5.4)	52 (3.9)	19 (5.0)	14 (3.1)	66 (11.8)	24 (3.2)	43 (2.9)	9 (2.5)	20 (4.5)	5 (0.9)	22 (3.1)	105 (11.6)	23 (1.8)	0 (0.0)

IQR: interquartile range

* at baseline

** 5 years before

*** 10 years before

A total of 73 different chronic condition categories with > 2% prevalence were identified at baseline, 56 categories were obtained five years before and 32 categories ten years before baseline. Figure S1 shows the prevalence of the selected chronic condition categories along the three time points. The most prevalent diagnoses all along the three time points were essential hypertension and osteoarthritis, followed by other conditions such as urinary incontinence, neurocognitive disorders, obesity or diabetes mellitus.

Five clusters of chronic multimorbidity were identified at baseline in patients with two or more chronic conditions. These clusters were labelled as follows: *Heart diseases*, *Metabolic and vascular diseases*, *Neurocognitive*, *Musculoskeletal and chronic pain syndrome* and *Unspecific*. Similar clusters were found in the time point defined 5 years before; therefore, the same labels were kept. Clusters that were assigned the same label in different time points presented different disease prevalence and presence but overall maintained similar over-represented diagnoses. Six clusters were identified 10 years before and were assigned the following labels: *Metabolic and vascular diseases*, *Male-predominant diseases*, *Minor conditions and sensory impairment*, *Musculoskeletal and chronic pain syndrome*, *Lipid metabolism disorders* and *Unspecific*. All in all, two clusters were found in all of the time points: *Metabolic and vascular diseases* and *Musculoskeletal and chronic pain syndrome*. Prevalence, O/E ratio and exclusivity of all chronic conditions used to define these multimorbidity clusters can be found in Tables S1, S2 and S3.

Membership probabilities of patients to each set of clusters per time point were calculated to describe the possible overlap between clusters (Fig S2). Most patients were found most probably assigned to a certain cluster. Median probability of the most probable cluster in patients at baseline was 99%, while it decreased to 87% and 76% at the time points of 5 years and 10 years before, respectively. Cluster membership became more defined at the later time points, suggesting that a higher disease burden lead to a more defined allocation to a certain cluster.

Descriptive statistics on the individuals’ number of chronic conditions, sex and age group according to the assigned multimorbidity cluster are shown in Table 1. Patients in the *heart diseases* clusters had the greatest number of chronic conditions (median was 13 conditions at baseline, 9 conditions five years before), while those in the *unspecific* clusters presented the lowest number (median was 6 conditions at baseline, 4 five years before and 3 ten years before). The *Musculoskeletal and chronic pain syndrome* clusters were those with highest presence of females in all three time points, while the *Male-predominant diseases* cluster had the highest prevalence of males but was only found in the earliest time point (10 years before baseline). Patients in the *No multimorbidity* group presented the largest proportion of youngest individuals (aged 66–70 at baseline) at each of the time points.

**Fig. 1:**
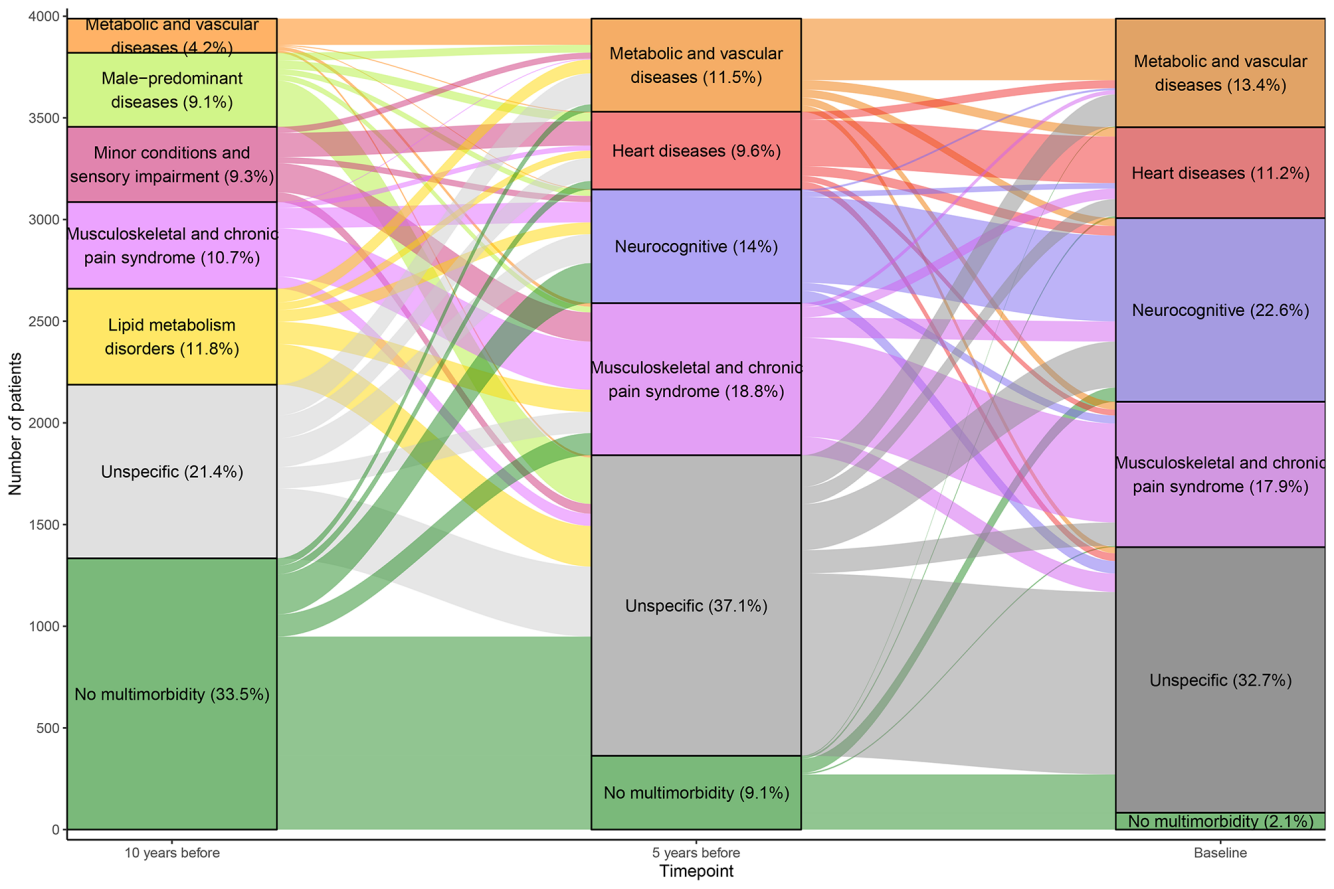
Prevalence of chronic multimorbidity clusters defined at three different time points over ten years and their trajectories. Bar heights represent the number of patients belonging to the cluster and stripe heights represent patients moving from one cluster to another, in an independent manner for each of the two depicted cluster transitions.

Retrospective trajectories of each patient were established along the three time points. Figure 1 shows all trajectories coloured according to the cluster of belonging in the previous time point, proportional to the number of patients transitioning from previous to next cluster. Different patterns of cluster transitioning between time points were found. In the first transition (10 to 5 years before), a variety of trajectories occurred and only 1031 patients (25.9%) transitioned to a similar cluster. Contrarily, in the second transition (5 years before to baseline), most patients (2047, 60.4%) transitioned to the same type of cluster. Frequencies of patients transitioning from clusters between two time points are shown in Figures S3A and B. Regarding entire identified trajectories, frequencies of the most prevalent ones can be found in Table S4. The top 3 complete trajectories involved non-multimorbid patients together with *unspecific* clusters, followed by a trajectory in which patients remained in the *musculoskeletal* cluster for 10 years and a trajectory in which patients transitioned from no multimorbidity to a *neurocognitive* cluster and remained there.

**Fig. 2:**
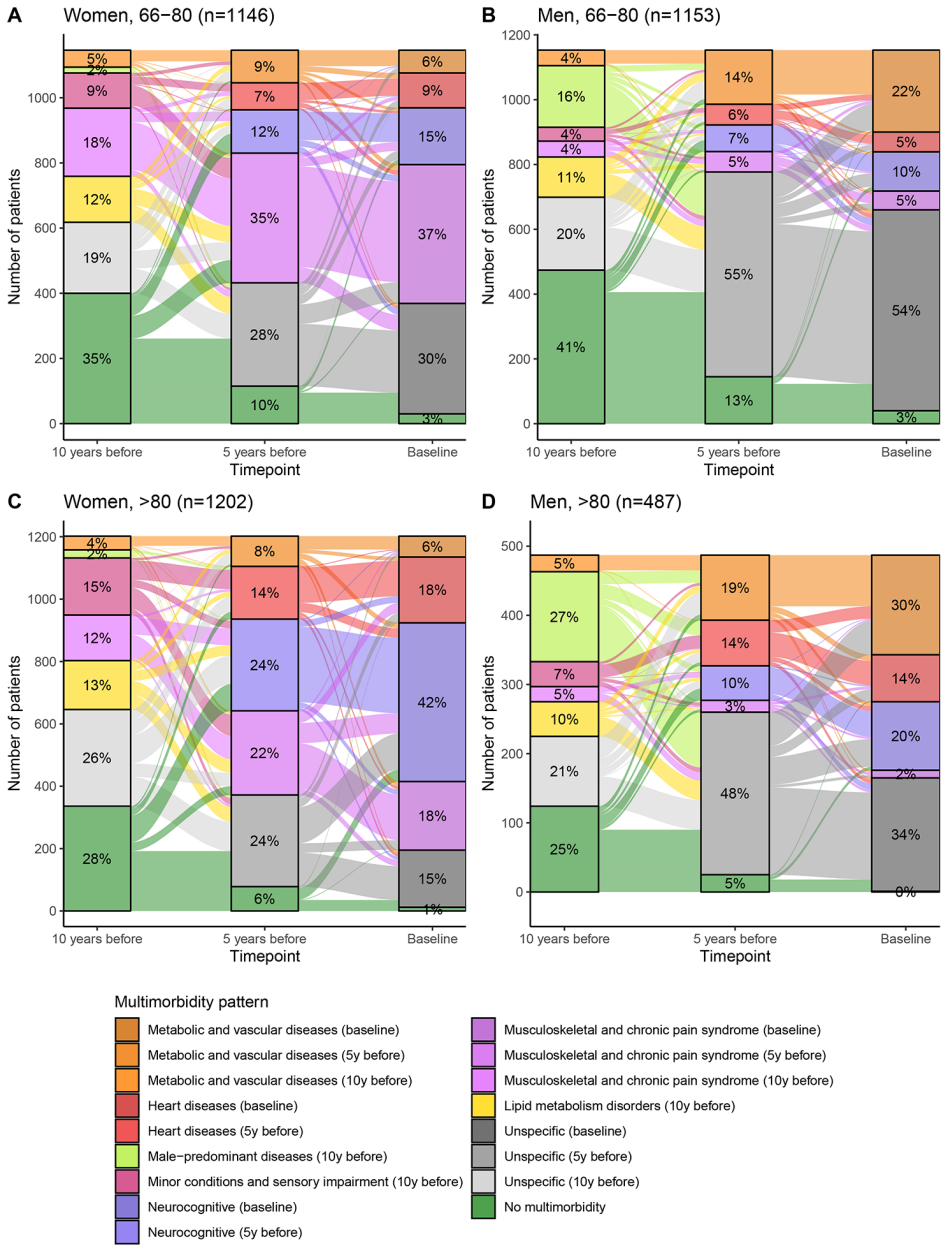
Prevalence of chronic multimorbidity clusters defined at three different time points over ten years and their trajectories, stratified by sex and age group. Bar heights represent the number of patients belonging to the cluster and stripe heights represent patients moving from one cluster to another, in an independent manner for each of the two depicted cluster transitions.

After describing the obtained clusters and trajectories, a stratification by sex and age group was conducted in order to uncover possible differences in those variables. Figure 2 shows the distribution of patients in the identified clusters of each stratum, as well as their trajectories. In males, the *Unspecific* cluster displayed the highest prevalence in both age groups, while in females, it was the *Musculoskeletal and chronic pain syndrome* cluster for the youngest and the *Neurocognitive* cluster for the oldest.

## Discussion

### Main important results

The findings of this study expand our understanding of the progression in chronic conditions among older patients by evaluating patterns of chronic multimorbidity and their trajectories over a 10-year period. Multimorbidity increased from 73.6 to 98.3% in this time span and the median number of chronic conditions progressed from 3 to 9. Two clusters emerged, named *Heart diseases* and *Neurocognitive*, along with the discontinuation of clusters likely requiring lower levels of disease management, revealing a substantial increase in multimorbidity burden. Furthermore, the differences uncovered by the sex and age stratification suggest that these variables should not be overlooked when planning and designing future actions. All in all, these findings highlight the dynamism and variation of multimorbidity.

### Comparison with other studies

This study explores and contributes to shed light on the trajectories of chronic multimorbidity patterns– i.e. how do patterns of diseases change over time and how do patients transition within these patterns. While several studies have already been exploring trajectories of multimorbidity with various methodologies [25], very few have determined comprehensive multimorbidity patterns with comparable techniques.

Guisado-Clavero and colleagues [31] analysed multimorbidity patterns in patients aged > 65 residing in Barcelona (Spain) in a 6-year span. The obtained clusters were found to remain quite similar from the beginning to the end of the study period and retain most patients, similar to our findings comparing our baseline time point to 5 years before. Moreover, from the six identified patterns (named *Musculoskeletal, Endocrine-metabolic, Digestive-respiratory, Cardiovascular, Neuropsychiatric* and *Unspecific*), five of them could be matched to those identified in our analyses. Another study, considering a longer period (12 years) in patients aged ≥ 60 from a Swedish city [32], found highly heterogeneous trajectories from the 6 initially identified multimorbidity patterns (named *Psychiatric and respiratory, Heart, Respiratory and musculoskeletal, Cognitive and sensory impairment*, *Eye diseases and cancer, Unspecific*) to those identified 12 years later (named *Vascular*, *Cardiometabolic*, *Respiratory*, *Neuropsychiatric*, *Eye and Musculoskeletal*, *Unspecific*). Therefore, similar results have been found in terms of identifying multimorbidity patterns, as most of them may be equivalent to ours; however, the results on transitions or trajectories of multimorbidity need to be further studied.

### Clinical interpretation of the results

The most common trajectory involving specific clusters was that of patients remaining in the *Musculoskeletal and chronic pain syndrome* cluster all along. This cluster consistently had the highest proportion of females (more than 80%), and not only showed a high prevalence of musculoskeletal and pain-related disorders but also of anxiety and depression. These findings are not surprising, as it is well-known that these conditions are frequent, more common in women, and increase with age [33–35]. Furthermore, this situation represents a significant health burden and may cause a highly negative impact on many aspects of life. Therefore, strong efforts should be directed towards development of better prevention and management strategies to address the complexity of this multimorbidity pattern. In this sense, new approaches are being proposed, such as the creation of highly specialised, interdisciplinary units that consider all aspects involved in the inflammatory vicious cycle of musculoskeletal pain in order to prevent its self-perpetuity and chronicity [36].

The next cluster showing a high consistency across all time points was the so called *Metabolic and vascular diseases*, mainly characterized by diabetes mellitus and peripheral/visceral vascular disease. It was also the specific cluster with the highest proportion of males in the baseline and 5 years before time points. In the earliest time point, this was the *Male-predominant diseases* cluster, with a high prevalence of chronic obstructive pulmonary disease. These findings may be explained as an effect of cardiovascular risk factors, especially tobacco consumption, which could be the focus of preventive actions [37].

Conversely, the *Heart diseases* and *Neurocognitive* clusters emerged in the second time point of the study. The former was characterized by heart failure, valve disorders or pulmonary heart disease, and the latter composed by neurocognitive disorders or frailty. These conditions have an increasing prevalence with age, so that our findings are coherent. Furthermore, it may be possible that these clusters emerged in our retrospective analysis and were not found in the earliest time point because they might have conferred a higher mortality risk. In fact, a recent study described a higher mortality of individuals in clusters characterized by cardiovascular and neuropsychiatric diseases compared to an unspecific cluster [32]. Moreover, some studies also show how these patterns may be strong contributors to physical decline and disability [38]. All in all, these clusters may encompass those most burdensome, age-dependent disorders in which the potential to reduce disease burden is proposed to come from primary, secondary and tertiary prevention targeting older people and not only middle-aged adults [39]. Thus, it may also be important to explore the inclusion of further clinical variables that might impact patient management such as geriatric syndromes, frailty or drug prescriptions to the analysis and definition of multimorbidity patterns [40–42].

Finally, the *Unspecific* cluster was characterized by a lack of overrepresentation of any chronic conditions so that the association between diseases could have happened by chance. It was composed of cardiovascular risk factors, osteoarthritis or vision impairment, among others. While most patients moved from unspecific to specific clusters, some patients moved from specific to unspecific. In fact, most patients from the *Male-predominant diseases* and the *Lipid metabolism disorders* clusters, moved to the *Unspecific* cluster at the first transition. This situation might be explained by the fact that the reciprocal relationship between diseases changed as a result of participants gradually accumulating new diseases. This resulted in some clusters no longer appearing in the analyses and a possibly higher heterogeneity in the unspecific cluster as age advances. Another important consideration on the *Unspecific* cluster is the large proportion of male individuals, specially aged > 80, present in this cluster, which might be explained by the selection of those healthier oldest individuals.

### Strengths and limitations

This study has several strengths. First, the inclusion of an exhaustive set of chronic condition diagnoses in the analyses, which allowed to define a set of comprehensive multimorbidity patterns. For instance, the inclusion of both mental and physical conditions enabled to describe their potential interplay. Second, the robust statistical methodology applied, fuzzy c-means clustering, which allowed to cluster individuals according to their co-occurring conditions and follow their trajectories over time. This is the choice method when there is a tendency of overlap in clusters, which may be frequent in older individuals with highly prevalent conditions. Third, the involvement of a multidisciplinary team in the consensus process of defining the multimorbidity clusters, which provides both statistical and clinical validity. Finally, considering a sex perspective in the stratified analysis, allowed to uncover possible differences between men and women which may help increase gender equity.

Nevertheless, some limitations of this work should also be considered. Despite comprehensively considering chronic conditions, some factors such as frailty, geriatric syndromes, chronic medication or care received could be relevant but are not available. However, this does not invalidate the novel methodological approach. In addition, the unavailability of hospital diagnoses could have introduced possible biases in the data. Nevertheless, the gatekeeping role general practitioners at primary care could be a compensatory mechanism. Another limitation would be the retrospective nature of the study. Therefore, it does not allow to account for trajectories of individuals who did not survive at ten years’ times.

Furthermore, the study cohort, composed of COVID-19 cases, might be introducing a bias in the estimated prevalences of chronic conditions in each cluster as well as in the frequencies of patients in each cluster, that may not reflect those of the general population However, taking advantage of this cohort, it is valuable to assess how the trajectories have developed in these patients. Moreover, some limitations are also present regarding the use of RWD from electronic health records, such as unavailability of certain variables or missing information. However, these databases guarantee maximum representation, large patient volumes and detailed information registered with relatively homogeneous criteria.

### Possible clinical implications

Multimorbidity is currently challenging the traditional approach of medicine, from clinical practice of professionals treating individual patients to management decisions of policy makers in charge of the organization of entire healthcare services. In this context, suggestions are being made to create integrated programs that connect various clinical specialties and healthcare units, with a primary focus on individual patients, their unique clinical profiles and trajectories. Hence, adopting a longitudinal perspective and considering multimorbidity patterns may contribute to this needed redefinition and reorientation of healthcare delivery towards multimorbid patients.

The findings in this study may help in the development of higher personalised medicine in multimorbidity and could also potentially be used to promote healthier aging. The stratification by sex and age allowed to identify possible clusters and trajectories on which some actions could be focused in order to define specific clinical protocols, prevention strategies, reorganization of healthcare circuits or planning for future needs. Thus, our findings support the design of future randomized clinical studies aimed at improving the clinical management of multimorbidity.

Moreover, this study is a contribution to P4 (predictive, personalised, preventive, and participatory) medicine, which is based on the analysis of large amounts of data, the use of artificial intelligence assistance and the organization of multidisciplinary teams to bring more efficient care to geriatric patients. All in all, both clinicians managing co-occurring chronic conditions and health policy makers allocating resources for care may benefit from understanding how diseases cluster together and, moreover, how multimorbidity might progress over time.

## Conclusions

Trajectories of chronic multimorbidity patterns in older patients are identifiable and show a high level of complexity and fluctuation over time. In a cohort of older patients (65 + years old), we were able to identify multimorbidity patterns of chronic conditions and describe their individual trajectories in the previous 10 years using RWD and cluster analysis. Taken together, our results suggest that, while further research is needed to develop a deeper understanding, considering multimorbidity patterns and their trajectories, along with incorporating a sex perspective, might improve decisions in clinical management and healthcare planning.

### Electronic supplementary material

Below is the link to the electronic supplementary material.

Supplementary Material 1 (Figure S1)

Supplementary Material 2 (Table S1)

Supplementary Material 3 (Table S2)

Supplementary Material 4 (Table S3)

Supplementary Material 5 (Figure S2)

Supplementary Material 6 (Table S4)

Supplementary Material 7 (Figure S3)

## Data Availability

Data cannot be publicly shared because of confidentiality. Data used for this analysis are available from the Government of Catalonia Institutional Data Access (PADRIS Program) for researchers who meet the criteria for access to confidential data. Inquiries of access to aggregated data should be addressed to AQuAS director (direccio.aquas@gencat.cat), under the following conditions: http://aquas.gencat.cat/ca/ambits/analitica-dades/padris/.

## Abbreviations

RWD: Real-world data
O/E: observed/expected
