# Supplementary material for: Trajectories of chronic multimorbidity patterns in older patients: MTOP study
Source: BMC Geriatr. 2024 May 30;24:475. doi: 10.1186/s12877-024-04925-2 (PMC11137950; doi:10.1186/s12877-024-04925-2)
Supplement: Supplementary file 1 — Supplementary Material 1 (Figure S1) [file 12877_2024_4925_MOESM6_ESM.pdf]

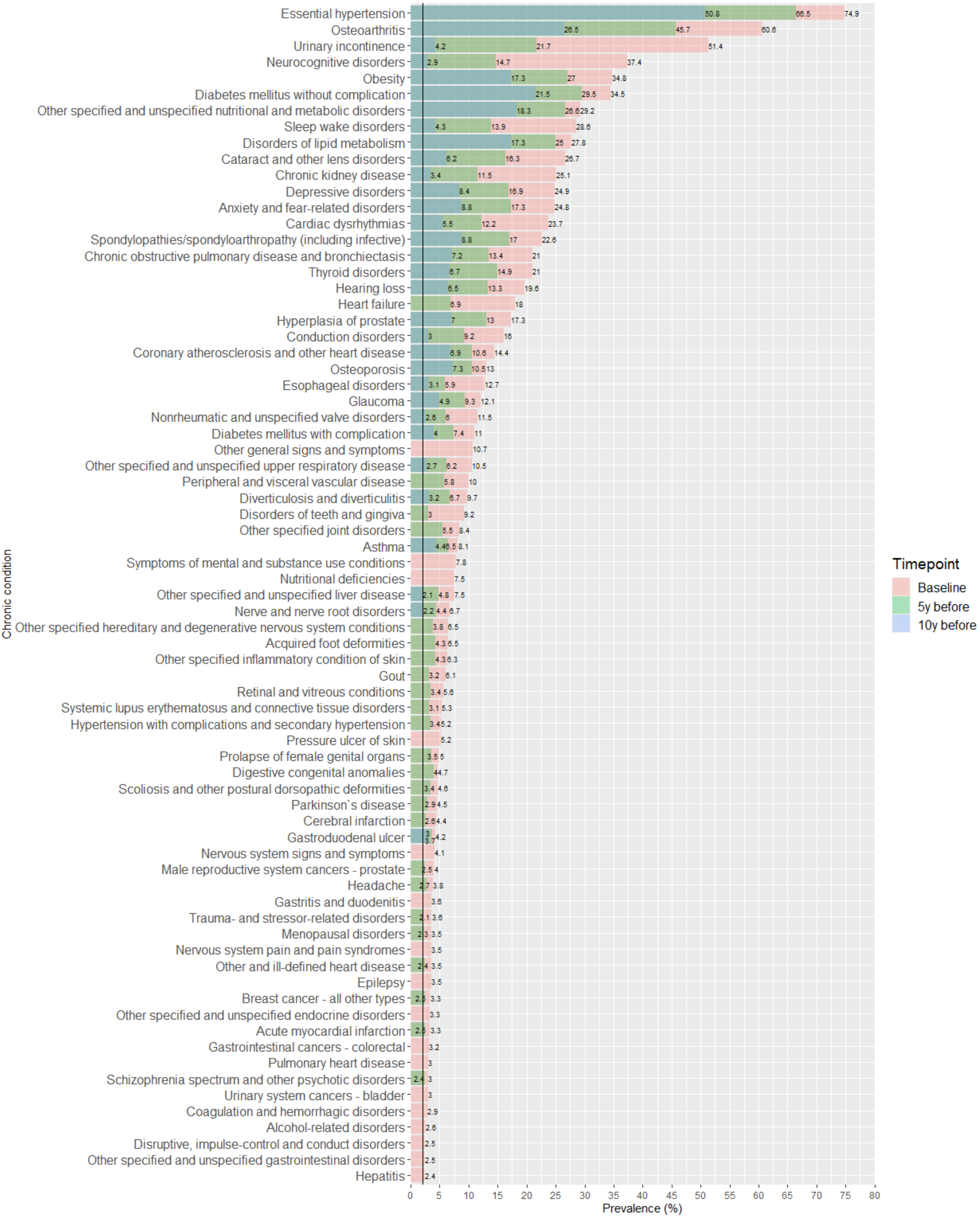

Figure S1. Prevalence of each chronic condition in the three time points analysed (baseline, 5 years before, 10 years before).
Chronic conditions were identified and grouped according to Chronic Condition Indicator and Chronic Condition Classification, filtered for >2% prevalence and ordered by prevalence at baseline.
