# Supplementary material for: Trajectories of chronic multimorbidity patterns in older patients: MTOP study
Source: BMC Geriatr. 2024 May 30;24:475. doi: 10.1186/s12877-024-04925-2 (PMC11137950; doi:10.1186/s12877-024-04925-2)
Supplement: Supplementary file 5 — Supplementary Material 5 (Figure S2) [file 12877_2024_4925_MOESM7_ESM.pdf]

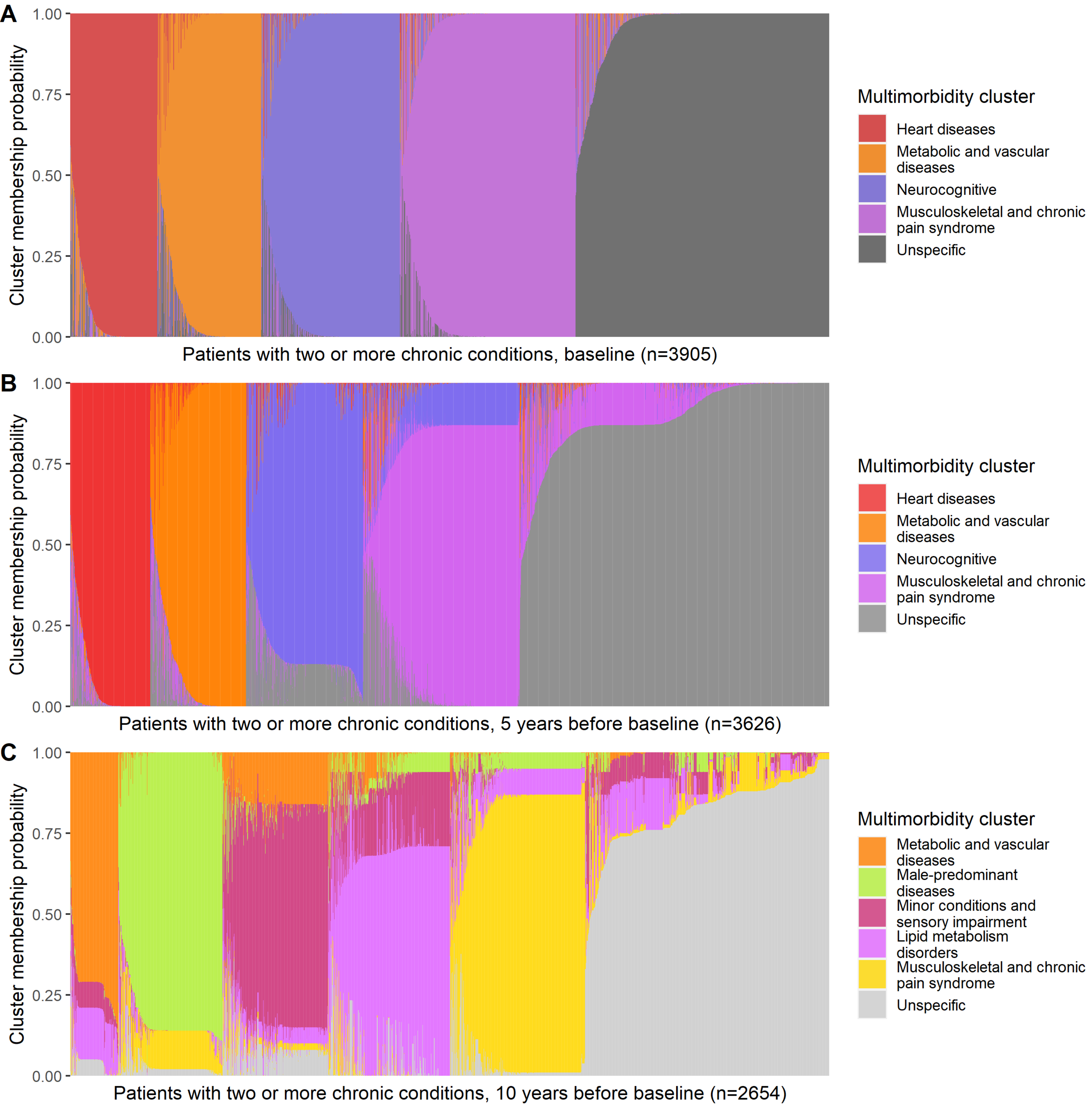

Figure S2. Distribution of multimorbidity cluster membership probabilities per patient (each column represents a patient) at the three defined time points. A: baseline. B: 5 years before baseline. C: 10 years before baseline.
