# Supplementary material for: Trajectories of chronic multimorbidity patterns in older patients: MTOP study
Source: BMC Geriatr. 2024 May 30;24:475. doi: 10.1186/s12877-024-04925-2 (PMC11137950; doi:10.1186/s12877-024-04925-2)
Supplement: Supplementary file 6 — Supplementary Material 6 (Table S4) [file 12877_2024_4925_MOESM1_ESM.docx]

| **10 years before** | **5 years before** | **Baseline** | **n** | **%** |
| --- | --- | --- | --- | --- |
| No multimorbidity | Unspecific | Unspecific | 398 | 9.98 |
| No multimorbidity | No multimorbidity | Unspecific | 188 | 4.71 |
| Unspecific | Unspecific | Unspecific | 184 | 4.61 |
| Musculoskeletal & chronic pain synd. | Musculoskeletal & chronic pain synd. | Musculoskeletal & chronic pain synd. | 169 | 4.24 |
| No multimorbidity | Neurocognitive | Neurocognitive | 151 | 3.79 |
| Male-predominant diseases | Unspecific | Unspecific | 135 | 3.39 |
| Lipid metabolism disorders | Unspecific | Unspecific | 118 | 2.96 |
| Metabolic & vascular diseases | Metabolic & vascular diseases | Metabolic & vascular diseases | 100 | 2.51 |
| Unspecific | Neurocognitive | Neurocognitive | 100 | 2.51 |
| Minor conditions & sensory impairm. | Musculoskeletal & chronic pain synd. | Musculoskeletal & chronic pain synd. | 98 | 2.46 |
| Unspecific | Metabolic & vascular diseases | Metabolic & vascular diseases | 93 | 2.33 |
| Minor conditions & sensory impairm. | Heart diseases | Heart diseases | 89 | 2.23 |
| No multimorbidity | No multimorbidity | No multimorbidity | 82 | 2.06 |
| Musculoskeletal & chronic pain synd. | Neurocognitive | Neurocognitive | 78 | 1.96 |
| No multimorbidity | Unspecific | Neurocognitive | 78 | 1.96 |
| No multimorbidity | No multimorbidity | Neurocognitive | 70 | 1.76 |
| No multimorbidity | Musculoskeletal & chronic pain synd. | Musculoskeletal & chronic pain synd. | 70 | 1.76 |
| Unspecific | Unspecific | Neurocognitive | 67 | 1.68 |
| Lipid metabolism disorders | Musculoskeletal & chronic pain synd. | Musculoskeletal & chronic pain synd. | 65 | 1.63 |
| Unspecific | Heart diseases | Heart diseases | 62 | 1.55 |
| Unspecific | Musculoskeletal & chronic pain synd. | Musculoskeletal & chronic pain synd. | 60 | 1.50 |
| Male-predominant diseases | Unspecific | Metabolic & vascular diseases | 44 | 1.10 |
| No multimorbidity | Unspecific | Metabolic & vascular diseases | 44 | 1.10 |
| Lipid metabolism disorders | Neurocognitive | Neurocognitive | 43 | 1.08 |
| Lipid metabolism disorders | Metabolic & vascular diseases | Metabolic & vascular diseases | 40 | 1.00 |
| No multimorbidity | Unspecific | Musculoskeletal & chronic pain synd. | 40 | 1.00 |
| Musculoskeletal & chronic pain synd. | Unspecific | Unspecific | 40 | 1.00 |
| Unspecific | Unspecific | Metabolic & vascular diseases | 39 | 0.98 |
| No multimorbidity | Neurocognitive | Unspecific | 31 | 0.78 |
| Male-predominant diseases | Metabolic & vascular diseases | Metabolic & vascular diseases | 30 | 0.75 |
| Unspecific | Unspecific | Musculoskeletal & chronic pain synd. | 30 | 0.75 |
| Lipid metabolism disorders | Unspecific | Neurocognitive | 29 | 0.73 |
| Musculoskeletal & chronic pain synd. | Musculoskeletal & chronic pain synd. | Neurocognitive | 28 | 0.70 |
| No multimorbidity | Unspecific | Heart diseases | 26 | 0.65 |
| Male-predominant diseases | Unspecific | Neurocognitive | 26 | 0.65 |
| Minor conditions & sensory impairm. | Neurocognitive | Neurocognitive | 25 | 0.63 |
| Unspecific | Unspecific | Heart diseases | 24 | 0.60 |
| No multimorbidity | Musculoskeletal & chronic pain synd. | Unspecific | 24 | 0.60 |
| Musculoskeletal & chronic pain synd. | Musculoskeletal & chronic pain synd. | Unspecific | 24 | 0.60 |
| No multimorbidity | Metabolic & vascular diseases | Metabolic & vascular diseases | 23 | 0.58 |
| Minor conditions & sensory impairm. | Musculoskeletal & chronic pain synd. | Neurocognitive | 22 | 0.55 |
| Lipid metabolism disorders | Heart diseases | Heart diseases | 21 | 0.53 |
| No multimorbidity | Heart diseases | Heart diseases | 21 | 0.53 |
| Male-predominant diseases | Neurocognitive | Neurocognitive | 21 | 0.53 |
| Lipid metabolism disorders | Unspecific | Metabolic & vascular diseases | 20 | 0.50 |
| Unspecific | Metabolic & vascular diseases | Heart diseases | 19 | 0.48 |
| Male-predominant diseases | Heart diseases | Heart diseases | 19 | 0.48 |
| Unspecific | Heart diseases | Neurocognitive | 19 | 0.48 |
| Minor conditions & sensory impairm. | Unspecific | Unspecific | 19 | 0.48 |
| Lipid metabolism disorders | Unspecific | Musculoskeletal & chronic pain synd. | 18 | 0.45 |
| Lipid metabolism disorders | Musculoskeletal & chronic pain synd. | Unspecific | 18 | 0.45 |
| Unspecific | Musculoskeletal & chronic pain synd. | Neurocognitive | 17 | 0.43 |
| Unspecific | Metabolic & vascular diseases | Neurocognitive | 16 | 0.40 |
| Lipid metabolism disorders | Musculoskeletal & chronic pain synd. | Neurocognitive | 16 | 0.40 |
| Minor conditions & sensory impairm. | Metabolic & vascular diseases | Metabolic & vascular diseases | 15 | 0.38 |
| Lipid metabolism disorders | Unspecific | Heart diseases | 15 | 0.38 |
| Unspecific | Neurocognitive | Heart diseases | 14 | 0.35 |
| Musculoskeletal & chronic pain synd. | Musculoskeletal & chronic pain synd. | Heart diseases | 14 | 0.35 |
| Unspecific | Musculoskeletal & chronic pain synd. | Heart diseases | 14 | 0.35 |
| Minor conditions & sensory impairm. | Unspecific | Neurocognitive | 14 | 0.35 |
| Male-predominant diseases | Musculoskeletal & chronic pain synd. | Musculoskeletal & chronic pain synd. | 14 | 0.35 |
| Unspecific | Neurocognitive | Unspecific | 14 | 0.35 |
| Musculoskeletal & chronic pain synd. | Heart diseases | Heart diseases | 13 | 0.33 |
| Unspecific | Heart diseases | Metabolic & vascular diseases | 12 | 0.30 |
| Metabolic & vascular diseases | Metabolic & vascular diseases | Heart diseases | 12 | 0.30 |
| Minor conditions & sensory impairm. | Musculoskeletal & chronic pain synd. | Heart diseases | 12 | 0.30 |
| Male-predominant diseases | Unspecific | Heart diseases | 12 | 0.30 |
| Unspecific | Metabolic & vascular diseases | Musculoskeletal & chronic pain synd. | 12 | 0.30 |
| Unspecific | Metabolic & vascular diseases | Unspecific | 12 | 0.30 |
| Musculoskeletal & chronic pain synd. | Unspecific | Neurocognitive | 11 | 0.28 |
| Musculoskeletal & chronic pain synd. | Neurocognitive | Musculoskeletal & chronic pain synd. | 11 | 0.28 |
| Unspecific | Neurocognitive | Musculoskeletal & chronic pain synd. | 11 | 0.28 |
| Metabolic & vascular diseases | Musculoskeletal & chronic pain synd. | Musculoskeletal & chronic pain synd. | 11 | 0.28 |
| Male-predominant diseases | Unspecific | Musculoskeletal & chronic pain synd. | 11 | 0.28 |
| Unspecific | Heart diseases | Unspecific | 11 | 0.28 |

Table S4. Topmost frequent chronic multimorbidity cluster trajectories across the three defined time points (10 years before, 5 years before, baseline), filtered up to 90% cumulative frequency.
