# Supplementary material for: Trajectories of chronic multimorbidity patterns in older patients: MTOP study
Source: BMC Geriatr. 2024 May 30;24:475. doi: 10.1186/s12877-024-04925-2 (PMC11137950; doi:10.1186/s12877-024-04925-2)
Supplement: Supplementary file 7 — Supplementary Material 7 (Figure S3) [file 12877_2024_4925_MOESM4_ESM.pdf]

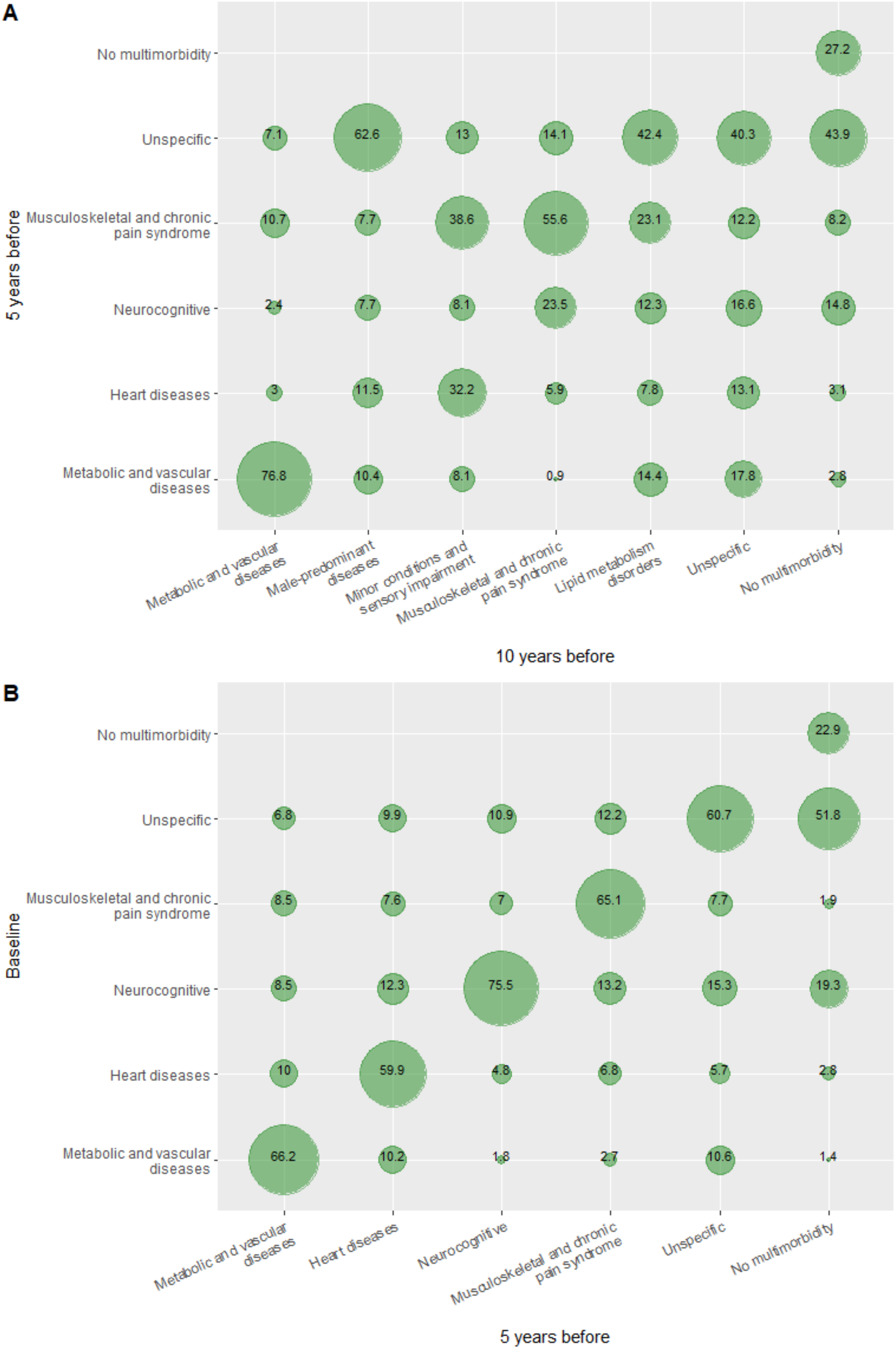

Figure S3. Proportion of patients transitioning from one cluster to another between time points.
A: 10 years before baseline versus 5 years before baseline time points (column %). B: 5 years before baseline versus baseline time points (column %).
